# Pro-Inflammatory Profile of Children Exposed to Maternal Chikungunya Virus Infection during the Intrauterine Period: A One-Year Follow-Up Study

**DOI:** 10.3390/v14091881

**Published:** 2022-08-26

**Authors:** Renan Faustino, Fabiana Rabe Carvalho, Thalia Medeiros, Débora Familiar-Macedo, Renata Artimos de Oliveira Vianna, Paulo Emílio Côrrea Leite, Isabela Resende Pereira, Claudete Aparecida Araújo Cardoso, Elzinandes Leal De Azeredo, Andrea Alice Silva

**Affiliations:** 1 Multiuser Laboratory for Research Support in Nephrology and Medical Sciences (LAMAP), Hospital Universitario Antonio Pedro, Faculty of Medicine, Universidade Federal Fluminense, Niteroi 24033-900, Brazil; 2 Department of Pathology, Faculty of Medicine, Universidade Federal Fluminense, Niterói 24033-900, Brazil; 3 Viral Immunology Laboratory, Oswaldo Cruz Institute, Fundação Oswaldo Cruz, Rio de Janeiro 21040-360, Brazil; 4 Clinical Research Unit (UPC), Universidade Federal Fluminense, Niterói 24033-900, Brazil; 5 Department of Maternal and Child, Faculty of Medicine, Universidade Federal Fluminense, Niterói 24033-900, Brazil

**Keywords:** Chikungunya, inflammation, maternal

## Abstract

Chikungunya virus (CHIKV) vertical transmission occurs due to maternal viremia in the prepartum. Clinical presentation in neonates can be varied; however, the consequences of intrauterine exposure on the immune response are unclear. Thus, we aimed to analyze inflammatory alterations in children exposed to maternal CHIKV infection. This is a cross-sectional study that included children exposed to maternal CHIKV infection (confirmed by RT-qPCR and/or IgM). Circulant immune mediators were analyzed by a multiplex assay. RESULTS: We included 33 children, with a mean age of 3 ± 2.9 months-old, and 19 (57.6%) were male. Only one child presented neurological alterations. CHIKV-exposed infants showed elevated levels of MIP-1α, MIP-1β, and CCL-2 (*p* < 0.05). Pro-inflammatory cytokines such as TNFα, IL-6, and IL-7 (*p* < 0.0001) were also increased. In addition, lower levels of PDGF-BB and GM-CSF were observed in the same group (*p* < 0.0001). Principal component (PC) analysis highlighted a distinction in the inflammatory profile between groups, where PC explained 56.6% of the alterations. Our findings suggest that maternal exposure to CHIKV can affect the circulating levels of pro-inflammatory cytokines during the infants’ first year of life. The long-term clinical consequences of these findings should be investigated.

## 1. Introduction

Chikungunya virus (CHIKV) is an Alphavirus of the *Togaviridae* family, approximately 70 nm in diameter. Its genetic material is composed of positive-sense single-stranded RNA with 11–12kb and has the hematophagous arthropods *Aedes Aegypti* and *Albopictus* as vectors [1,2]. Chikungunya fever (CHIKVF) quickly became endemic in Latin America, becoming an important public health problem. In Brazil, climatic and sanitary conditions facilitate *Aedes* spp. proliferation and, consequently, the spread of arboviruses [1,3]. Between 2014 and 2021, more than 1 million cases were reported in Brazil [4]. Published data shows that case fatality rate is approximately 2–5% [5,6].

CHIKV genome has two open reading frames (ORFs). The 5′ ORF encodes four non-structural proteins (nsP1, nsP2, nsP3 and nsP4) and the 3′ encodes four structural proteins (E1, E2, E3, C) [2]. The E2 subunit of the E protein promotes viral entry into host cells by mainly binding with the Mxra8 receptor and inducing a clathrin-mediated endocytosis. Mxra8 is a cell receptor that enhances the ability of CHIKV and other *Alphaviruses* to infect skeletal muscle cells and fibroblasts, being strongly associated with musculoskeletal pathogenesis [7,8]. Asian, East/Central/South African (ECSA) and West African are the main genotypes of CHIKV that differ from one other in the E protein expression. In addition, a lineage descended from ECSA was identified: ECSA derived Indian Ocean lineage (IOL) [9]. Through phylogenetic analysis and molecular characterization of CHIKV representative strains, Alves-Souza and colleagues (2018) demonstrated the ECSA genotype circulation in Rio de Janeiro (RJ)-Brazil [10].

Clinical presentation can vary according to patient’s characteristics [11]. CHIKV infection can lead to acute and chronic symptoms with debilitating arthralgia and myalgia for months or years. Notably, disease severity is strongly associated with viral persistence in cells such as monocytes, which leads to the establishment of a proinflammatory state [12]. In this regard, an exacerbated production of pro-inflammatory mediators such as interleukins (IL) -6, -1β, tumor necrosis factor α (TNF-α), and monocyte chemo-attractant protein-1 (MCP-1) has been reported at different stages of the disease [13].

Other forms of CHIKV propagation have been described, including vertical transmission [14]. In pregnant women, CHIKV infection is not associated with fetal teratogenic effects; however, the higher risk of vertical transmission in the prepartum (7 days before birth) can lead to clinical manifestations in up to 90% of cases [14,15]. The newborns present low viremia or undetectable viral load at birth, although they may develop post-birth symptoms such as fever, rash, irritability, as well as central nervous system (CNS) complications [16,17]. In this context, many studies have described CHIKV in pediatric cohorts with autochthonous infection, but little is known about the effects of intrauterine exposure.

Thus, since disturbances in the maternal inflammatory status may directly affect fetal development through the vertical transfer of hormones, cytokines, chemokines, and chimeric cells [18]; we sought to investigate the inflammatory *milieu* of infants exposed to maternal CHIKV in association with clinical events. For this, our study focused on the assessment of circulating levels of immune mediators, in addition to clinical-laboratory parameters, during the clinical follow-up of these children.

## 2. Materials and Methods

### 2.1. Study Design and Data Collection

This study was approved by the National Research Ethics Commission (CONEP, Brazil; CAAE: 56913416.9.0000.5243) and was conducted according to the Helsinki declaration. All study procedures were performed after obtaining written consent from the children’s parents. 

We conducted a cross-sectional study at the Exanthematic Disease Unit of the Hospital Universitário Antônio Pedro (HUAP, Niterói, Brazil) from March 2017 to March 2019. Mothers who presented cutaneous rash during pregnancy and their children were examined by a multidisciplinary team [19]. Maternal CHIKV infection was confirmed by molecular (real-time reverse transcription–polymerase chain reaction-RT-PCR) and/or serology (IgM) assays, which were performed at the reference laboratory of Rio de Janeiro State, Central Public Health Laboratory Noel Nutels (LACEN, Rio de Janeiro, Brazil) [20,21].

### 2.2. Patients

This study included infants intrauterine exposed to maternal infection by CHIKV (CHIKV group). In the control group (CTR), we included children whose mothers presented cutaneous rash during pregnancy and negative RT-qPCR results or serology for arbovirus infection (CHIKV, Zika-ZIKV and dengue-DENV) and others congenital diseases (syphilis, toxoplasmosis, rubella, cytomegalovirus, and HIV infection). Moreover, we evaluated patients according to age groups: newborns (up to 28 days-old), 1–3 months-old, >3–6 months-old, and >6–12 months-old. 

### 2.3. Blood Sampling

For biochemical and hematological analysis, venous blood samples were collected in BD Vacutainer^®^ blood collection tubes with serum coagulation activator (Becton Dickson, NJ, USA) and BD Vacutainer^®^ with EDTA K2 anticoagulant (Becton Dickson, NJ, USA) to obtain serum and plasma, respectively. For the analysis of circulating immune mediators, serum was obtained by centrifugation (1210× *g*/10 min) at room temperature using the LS-3 Centrifuge^®^ (Celm, São Paulo, Brazil) and immediately stored at −80 °C until the experiments were carried out. All serology, biochemical and hematological tests were performed by the Service of Clinical Pathology (HUAP, UFF, Niterói, Brazil).

### 2.4. Multiplex Immunoassay

The Bio-Plex Magpix commercial kit (Biorad Laboratories Inc., Hercules, CA, USA) was used for assessing serum levels of immune mediators following the manufacturer’s recommendations. The magnetic “xMAP” technology allows the detection of 27 different circulating proteins based on microspheres. The following mediators were quantified: (i) chemokines = monocyte chemoattractant protein1 (MCP-1/CCL-2), macrophage inflammatory protein 1a (MIP-1a/CCL-3), inflammatory macrophage protein 1β (MIP-1β/CCL-4), linker 5 of the chemokine C-C RANTES (CCL-5), ligand 11 of the protein C-C Eotaxin (CCL-11), protein induced by interferon-γ 10 (IP-10/CXCL-10), include chemokine (C-X-C motif) ligand 8 (CXCL-8); (ii) growth factors = granulocyte-macrophage colony stimulating factor (G-CSF/CSF-2), granulocyte colony stimulating factor (G-CSF/CSF-3), basic fibroblast growth factor (FGFb), platelet-derived growth factor BB (PDGF-BB), vascular endothelial growth factor (VEFG); (iii) cytokines = interferon-γ (IFN-γ), interleukin 1 receptor antagonist (IL-Ra); interleukins (IL) 1β, -2, -4, -5, -6, -7, -9, -10, -12p70, -13, -15, -17 and tumor necrosis factor-ɑ (TNF-ɑ).

### 2.5. Detection of Anti-CHIKV IgM and IgG Antibodies 

CHIKV specific IgM and IgG immunoglobulins were semi-quantified in serum samples from infants intrauterine exposed to maternal CHIKV infection. The capture ELISAs assay were performed following the manufacturer’s protocol (Euroimmun, Lubeck, germany). Optical densities (ODs) were obtained according to the manufacturer’s instructions and the results were calculated in a semi-quantitative fashion using a ratio of samples ODs (or controls) divided by the calibrator’s OD. Samples with a result ≥ 1.1 were considered positive, as recommended by the manufacturer.

### 2.6. Statistical Analysis

Data was expressed as mean ± standard deviation (SD). For bivariate analysis between two independent groups, t-student or Mann–Whitney tests were used according to variable’s distribution. Analysis between three or more independent groups was performed by ANOVA or Kruskal–Wallis, with respective post-tests. We used the Statistical Package for the Social Sciences (SPSS Inc., Chicago, IL, USA) version 18.0 and GraphPad Prism version 8.0 (GraphPad Prism, San Diego, CA, USA) for statistical analysis. Values of *p* < 0.05 were considered significant.

To assess the population pattern and verify the interrelationship between the mediators, we performed a Principal Component Analysis (PCA). Data were analyzed using the Soft Independent Modelling by Class Analogy version 17.0 (SIMCA). Hotelling’s ellipse represents the 95% confidence interval and statistical significance follows the Rz rule. The R2x values represent the explanation rate of the variables by the modeling of the principal components.

## 3. Results

### 3.1. Study Population Characteristics 

From March 2017 to March 2019, 33 children exposed to maternal CHIKV infection were recruited. In addition, 14 infants were included in the CTR group. The frequency of males was slightly higher in both groups (around 57%). Two infants (6.1%) in the CHIKV group had clinical abnormalities: one (3%) presented neurodevelopmental delay and one (3%) presented postnatal sepsis. Audiological, ophthalmological, or cardiac abnormalities were not identified in any infants. Regarding the mother’s clinical presentation, maternal rash occurred more frequently in the third trimester (*n* = 14; 42.4%) in the CHIKV group. Arthralgia and myalgia were described in all cases of maternal CHIKV infection (*p* < 0.0001) and fever was reported in 26 cases (78.8%). None of the pregnant women in the CTR group presented joint pain; but four (28.6%) had myalgia. Demographic and clinical data of mothers and their children are described on Table 1.

When analyzing biochemical and hematological parameters, we did not observe significant alterations between CHIKV and CTR, except for serum C-reactive protein (*p* = 0.02). However, we identified that CHIKV patients presented a slight increase in serum ferritin (214.3 ± 219.0 vs. 89.5 ± 58.1), total bilirubin (1.5 ± 2.2 vs. 0.3 ± 0.1), indirect bilirubin (1.4 ± 2.1 vs. 0.2 ± 0.1), alanine aminotransferase (51.4 ± 46.1 vs. 41.1 ± 11.5) and aspartate aminotransferase (36.9 ± 46.0 vs. 25.1 ± 9.4) (Table 2). 

Regarding the serological parameters, we observed that all infants, both exposed to maternal CHIKV infection and CTR, did not present detectable CHIKV-specific IgM in the postnatal period. Of the 32 children evaluated in the CHIKV group, 25 (78.1%) were IgG+ and 7 (21.9%) were IgG−. Among the IgG− cases, four (12.5%) pregnant women were exposed in in the prepartum period.

### 3.2. Analysis of Circulant Inflammatory Mediators

**Figure 1:**
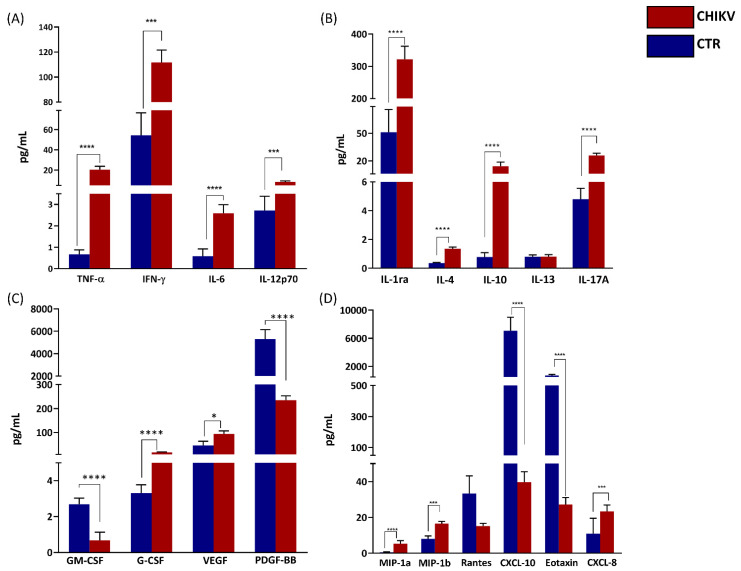
Children exposed in utero to maternal CHIKV infection present an exacerbated inflammatory environment. Quantification of circulating inflammatory mediators were performed by multiplex assay, including proinflammatory cytokines (**A**), anti-inflammatory cytokines (**B**), growth factors (**C**) and chemokines (**D**). Results are expressed as mean and standard deviation of each analyte. For statistical analysis, ANOVA or Kruskal–Wallis was used according to the normality of variables. CHIKV = Chikungunya. CTR = Control. *p* < 0.05 was considered significant. * *p* < 0.05; *** *p* < 0.001; ****p < 0.0001.

As demonstrated in Figure 1, the analysis of circulating immune mediators showed that significantly increased levels of pro-inflammatory cytokines such as TNF-α (*p* < 0.0001), IFN-γ (*p* = 0.002), IL-6 (*p* < 0.0001), and IL-12p70 (*p* < 0.0001); and anti-inflammatory cytokines such as IL-1Ra (*p* < 0.0001), IL-4 (*p* < 0.0001), IL-10 (*p* < 0.0001), and IL-17A (*p* < 0.0001) were observed in CHIKV group. Increased CCL-3 (*p* < 0.0001), CCL-4 (*p* = 0.0004), CXCL-8 (*p* = 0.0002), and decreased CXCL-10 (*p* < 0. 0001) and CCL-11 (*p* < 0.0001) were also observed in the same group. Among the growth factors, concentrations of VEGF (*p* = 0.0162) and G-CSF (*p* < 0.0001) were also higher in the CHIKV group. On the other hand, GM-CSF (*p* < 0.0001) and PDGF-BB (*p* < 0.0001) were significantly lower. Of note, serum levels of five cytokines (IL-1β, IL-2, IL-7, IL-9 and IL-15) were below the lower detection limit in all infants and were not analyzed (Figure 1). 

### 3.3. Principal Component Analysis (PCA) Shows the Relationship between Immune Mediators between Groups and According to Age and Gender

**Figure 2:**
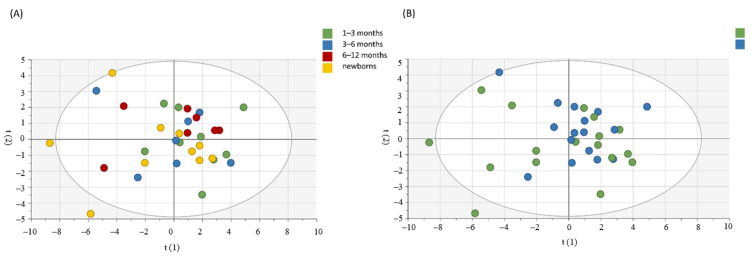
Principal component analysis for the assessment of influence of age (**A**) and gender (**B**) in the circulant levels of inflammatory mediators in children exposed to maternal CHIKV virus infection. The Hotelling ellipse represents the 95% confidence interval for the model.

**Figure 3:**
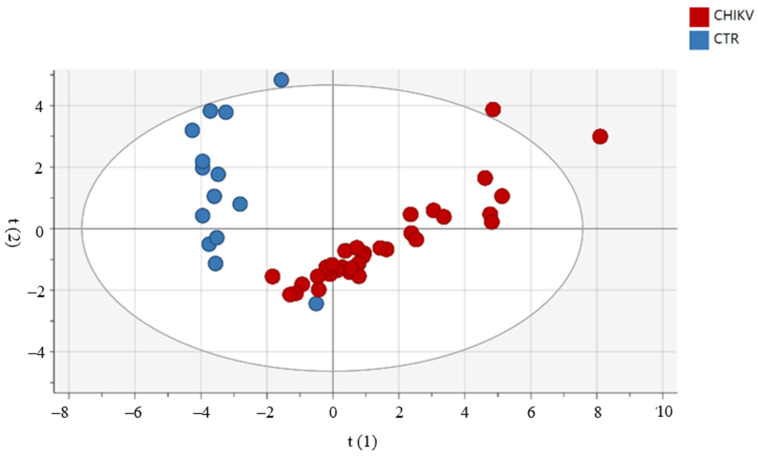
Principal component analysis reveals that the profile of inflammatory mediators in children exposed to maternal CHIKV infection is distinct from the control group. The *Hotelling* ellipse represents the 95% confidence interval for the model.

For the assessment of a possible influence of age and gender on circulating immune mediators in the CHIKV group, we used the PCA. As shown in Figure 2, there was no clear segregation between groups by age and gender. Moreover, we used PCA to observe patterns of inflammatory mediators between groups (CHIKV and CTRL). Confirming our data, the groups are distinct, as shown in Figure 3. We highlighted the mediators that best explain the variations by accumulated variables R2X (R2Xcum) and the mediators with greater predictability by the reliability index accumulated variables (Q2cum). IL-1Ra, TNF-α and IL-12 contributed to PC1 in children exposed to maternal CHIKV while in control group eotaxin and PDGF-BB influences in the PC2 analysis. R2X explained 54.8% of the alterations observed in the groups. In this sense, IL-1Ra (Q2VXcum = 0.64), IL-12p70 (Q2VXcum = 0.73) and PDGF-BB (Q2VXcum= 0.58) presented the best values of reliability. 

## 4. Discussion

Our findings showed an altered peripheral inflammatory profile in children exposed to maternal CHIKV infection. The impact of intrauterine exposure to maternal CHIKV infection is still unclear. It is known that congenital infectious diseases such as HIV, Influenza A and B, Herpes Simplex 1 and 2 can increase the risk of developing neurological alterations in the newborns, which can be associated with an immunological imbalance [18,22]. The first signs can appear at two years of age or even in adulthood; thus, monitoring possible hematological, biochemical and immunological changes due to viral infections in the first years of children exposed to maternal CHIKV can elucidate possible consequences in this particularly vulnerable population to inflammatory changes [18,23,24].

Concerning the analysis of inflammatory mediators, we observed that children in the CHIKV group presented higher levels of pro-inflammatory cytokines such as TNF-α, IL-6, and IFN-γ. These cytokines have distinct roles during neurodevelopment, inhibiting the proliferation and differentiation of neuronal cells [25,26]. In contrast, IFN-γ and IL-12p70 promote a neuroprotective role during fetal and infant development [22,26]. TNF-α is linked to neuroinflammation and, in high concentrations, has a neurotoxic and neurodegenerative capacity [26]. Moreover, TNF-α, IL-6, IL-1β, IFN-γ are also related to insulin resistance in the pediatric population [27]. Children in the CHIKV group also presented higher serum levels of chemokines, such as CXCL-8, MIP-1a and MIP-1b. Despite being constitutively produced, CXCL-8 is a potent chemokine that acts in chemotaxis, the regulation of apoptosis and the migration of neuronal cells [28,29,30]. Early or prenatal exposure to high levels of CXCL8 is associated with an increased risk for psychosis in adulthood [30]. The role of MIP-1a and MIP-1b in this phase remains uncertain, but MIP-1a has been reported to have a neuroprotective role when induced by umbilical cord monocytes [30]. Interestingly, children with vertically transmitted ZIKV infection presenting microcephaly showed elevated CXCL-10 levels and CXCL-9 in cerebrospinal fluid obtained after birth; however, in our study, only CXCL-10 concentrations were decreased [31]. Moreover, CXCL-10 in acute CHIKV and DENV infections are associated with neuroinvasive capacity and neuronal damage [32,33]. Eotaxin can inhibit the proliferation of neuronal progenitor cells [34]. Associated with other mediators such as IL-3, IL-1β; eotaxin was considered as a differential marker in children exposed in utero to ZIKV, born with or without microcephaly [35]. Low levels of CXCL10 and eotaxin observed in children exposed to maternal CHIKV infection may be associated with an infrequent neurological damage [32]. In fact, only one child exposed to maternal CHIKV infection presented alterations in the neurological clinical evaluation. Maybe it is necessary a direct contact with the virus in the gestational period to trigger neurological damage, or it could be revealed later in the child’s development.

We also identified elevated levels of anti-inflammatory cytokines in the CHIKV group, which could be a possible counter-regulatory mechanism in response to increased pro-inflammatory cytokines. A Th2 profile response (IL-4, IL-10, IL-13) in cases of CHIKV infection is associated with symptoms in the subacute phase (>14 days) and late resolution of musculoskeletal symptoms [36]. The strong activation of Th2 response in children, associated with other factors, is related to cases of atopic dermatitis. In topical dermatitis in infants, Th2 activation is accompanied by strong activation of the Th17 response [37]. Furthermore, the increase in IL-10 and other anti-inflammatory cytokines in the pediatric population is related to the development of allergic diseases such as rhinitis and asthma [38].

Recent studies reported that inflammatory disorders during the gestational period increase the risk of developmental changes during early childhood. Alterations can occur either by the vertical transfer of cells and inflammatory mediators that act systemically in the infant’s body for months or years or by changes in the epigenetic mechanisms of fetal programming, under the influence of the inflammatory microenvironment [18,39,40]. In addition, the transfer of immune mediators such as IL-10, MIP-1a, MIP-b, IL-1Ra and IL-4 can be promoted by placental extracellular vesicles [41]. Altogether, these data reinforce the importance of long-term clinical and laboratory follow-up of children presenting changes in the inflammatory *milieu* due to maternal viral infections, even in the absence of clinical abnormalities in the first year of life. The real impacts of the pro-inflammatory unbalance and exacerbation effect in this population will only be elucidated in the coming years when following the child’s development. 

Nevertheless, we observed one case (3.03%) of vertical CHIKV transmission, confirmed by positive RT-qPCR test in cerebrospinal fluid (data not shown). After the birth, the child developed encephalopathy and postnatal sepsis. Similarly, studies performed in children vertically infected by CHIKV reported delays in language development, speech and reasoning. [17,42,43]. Meanwhile, more recent data state that there are no abnormalities related to maternal CHIKV exposure apart from the intrapartum period up to two years of age. Research carried out in uninfected children exposed in utero to maternal HIV infection observed correlations between neurodevelopmental delay only at four years of age and an exacerbation of inflammatory mediators in these individuals [22]. Our group also studied children with vertically transmitted ZIKV infection with Congenital Zika Syndrome (CZS) and without CZS. We described proteomic alterations associated with the development of neurological diseases in the children without CZS that present an increased activity of metalloproteins 2 and 9 [24].

Some mediators may vary according to gender and age [44,45], so we performed PCA analysis to clarify this question. No cluster or significant data were found. The bivariate analysis also did not show notable differences. Apparently, the alterations observed in the CHIKV group are not related to these factors. Furthermore, this response appears to be sustained, as there were no differences between neonates and infants older than six months. A paired analysis was performed with a small randomly chosen group and we observed no significant differences between samples within a three-month interval of collection (Figure S1).

When comparing children exposed to CHIKV maternal infection and unexposed children through PCA, we confirmed that the populations are distinct. The drag towards the right observed in the children exposed to maternal CHIKV infection is mainly driven by the cytokines TNF-a, IL-1Ra and IL-12. This data supports our hypothesis of exacerbation of the inflammatory status of infants. Cytokines that have higher R2 and Q2 (>0.5) may be related to a higher risk of developing allergic and lung diseases, such as asthma and bronchitis, in addition to psoriasis and dermatitis [37]. On the other hand, PDGF-BB and eotaxin have a higher correlation with the unexposed children. Alterations in growth factors can impact neurodevelopment and angiogenesis and the maturation of other organs and systems [46].

Serological data converge with the literature, where children exposed to maternal CHIKV infection in the first trimester have a higher concentration of antibodies when compared to those exposed in the third trimester [47]. Therefore, this finding must be related to the longer contact time after maternal infection, allowing more significant IgG transfer to the fetus. In addition, we also evidenced the absence of IgM in all infants. Moreover, we selected child’s samples (*n* = 4) with high IgG concentrations presenting and performed a second collection after 18 months of age. We observed undetectable IgG levels (data not shown). One question to clarify is only the direct contact with CHIKV can stimulate the immune response. In vitro studies have shown that human syncytiotrophoblastic cells are not permissive to CHIKV [48,49]. However, recent studies identified the presence of CHIKV inside infected human syncytiotrophoblasts cells by antigen and PCR methods in placenta [50]. Thus, the possibility of children’s direct contact with the virus cannot be disregarded.

Regarding the analysis of other laboratory parameters, we observed no significant differences or relevant variations in routine biochemical and hematological tests. However, due to the impact that proteins can exert on several systems in the medium and long term, these findings discard future alterations [51]. Of note, some children of the CHIKV group presented serum ferritin > 600ng/dL despite the absence of clinical manifestations. Serum ferritin had been proposed in viral infection by IL-8 activation pathway [52] and as an earlier predictor of dengue fever severity, which may reach up to 750 ug/L [53]. 

This study has limitations. All children were exposed to maternal infection by CHIKV in utero; however, we were unable to confirm the vertical transmission by the placental analysis. Moreover, we were unable to associate inflammatory mediators with differences genotype, since only teh ECSA genotype circulates in Rio de Janeiro State [10]. Until the end of this study, no clinical alterations related to inflammatory unbalance have been observed, indicating the need for further longitudinal studies. In accordance with our results, we strongly recommend that infants are periodically monitored during early childhood, as the appearance of future manifestations cannot yet be ruled out.

## 5. Conclusions

Our results showed that children exposed to maternal CHIKV infection during the intrauterine period presented elevated circulating levels of inflammatory mediators during the first year of life, independently of age and sex. Furthermore, intrauterine exposure to CHIKV infection generates a distinct peripheral inflammatory *milieu*, mainly influenced by pro-inflammatory cytokines such as TNF-α and IL-12p70.

## Figures and Tables

**Table 1:** Demographic and clinical data of mothers infected with CHIKV during pregnancy and their intrauterine exposed infants recruited from 2017 to 2020.

Clinical Features	CHIK (*n* = 33)	Control (*n* = 14)	*p*-Value
Infants, *n* (%)			
Age (months. mean ± SD)	3.0 ± 2.9	9.9 ± 7.2	<0.0001
Male Gender	19 (57.0)	8 (57.1)	>0.9
Postnatal sepsis	1 (3.03)	0 (0)	0.9
Neurodevelopment disorders	1 (3.03)	0 (0)	0.9
Mothers, *n* (%)			
Age (year. mean ± SD)	26.8 ± 5.6	29.9 ± 6.2	>0.9
Time of maternal rash			
1th Trimester	6 (18.2)	3 (21.4)	0.7
2nd Trimester	13 (39.4)	6 (42.8)	0.7
3rd Trimester	14 (42.4)	5 (35.7)	>0.9
Clinical symptoms			
Fever	26 (78.8)	7 (50)	0.08
Myalgia	33 (100)	4 (28.6)	<0.0001
Arthralgia	33 (100)	7 (50)	<0.0001

Chi-square test were results of results of *p*-value less than 0.05. SD = Standard Deviation.

**Table 2:** Hematological and biochemical values of infants exposed intrauterine to CHIKV mother infection.

Parameters	Reference Range	CHIK (*n* = 33)	Control (*n* = 14)	*p*-Value
Alanine aminotransferase (U/L)	14.0–50.0	51.4 ± 46.1	41.1 ± 11.5	0.9
Aspartate aminotransferase (U/L)	15.0–37.0	36.9 ± 46.0	25.1 ± 9.4	0.3
Total Bilirrubin (mg/dL)	0.2–1.0	1.5 ± 2.2	0.3 ± 0.1	0.2
Direct Bilirrubin (mg/dL)	> 0.3	0.2 ± 0.1	0.1 ± 0.07	0.7
Indirect Bilirrubin (mg/dL)	-	1.4 ± 2.1	0.2 ± 0.1	0.06
Ferritin (ng/dL)	26–388.0	214.3 ± 219.0	89.5 ± 58.1	0.1
C-reactive Protein (mg/dL)	0.1–1	0.4 ± 0.1	0.1 ± 0.08	0.02
Lactate dehydrogenase (U/L)	85.0–227.0	327.2 ± 81.3	375.5 ± 173.9	0.5
Hemoglobin (g/dL)	11.0–14.0	11.8 ± 1.9	11.4 ± 3.3	0.05
Hematocrit (%)	33.0–39.0	35.2 ± 5.4	34.8 ± 9.0	0.1
Platelets (10^3^/mm^3^)	150.0–400.0	400.6 ± 166.4	477.2 ± 162.3	0.1
White blood cell count (10^3^/mm^3^)	5.0–17.0	11.1 ± 5.2	11.8 ± 4.6	0.8
Neutrophils	1–7	3.6 ± 1.9	3.7 ± 2.2	0.7
Lymphocytes	3.5–11	5.8 ± 1.9	5.4 ± 1.4	0.9
Monocytes	0.2–1	0.7 ± 0.4	0.9 ± 0.4	0.2
Eosinophils	0.1–1	0.3 ± 0.2	0.3 ± 0.1	0.2

Statistical analysis: Student’s *t*-test or Mann–Whitney test. Data are presented as mean ± SD. *p*-values < 0.05 were considered significant.

## Data Availability

Not applicable.

## Supplementary Materials

The following supporting information can be downloaded at: https://www.mdpi.com/article/10.3390/v14091881/s1, Figure S1: Paired analysis of children exposed in utero to maternal CHIKV infection. Samples of children in the first trimester of life (0–3 months) and second trimester (3–6 months).
