# Supplementary material for: Pro-Inflammatory Profile of Children Exposed to Maternal Chikungunya Virus Infection during the Intrauterine Period: A One-Year Follow-Up Study
Source: Viruses. 2022 Aug 26;14(9):1881. doi: 10.3390/v14091881 (PMC9501274; doi:10.3390/v14091881)
Supplement: Supplementary file 1 [file viruses-14-01881-s001.zip › viruses-1868001-supplementary.pdf]

Supplementary Materials:

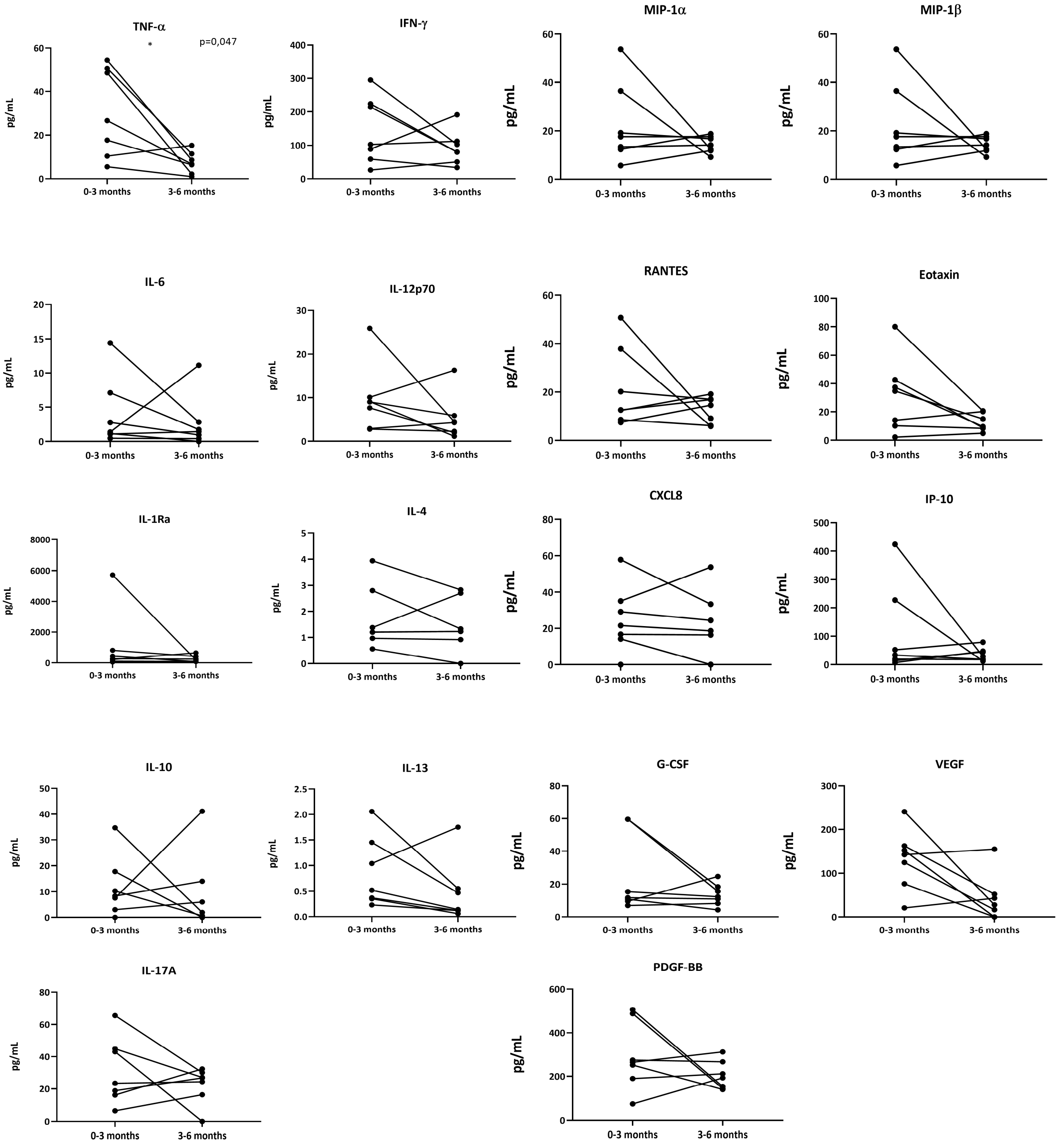

Figure S1. Paired analysis of children exposed in utero to maternal CHIKV infection. Samples of children in the first trimester of life (0–3 months) and second trimester (3–6 months). For statistical analysis, PAIRED T TEST or WILCOXON was used according to the normality of the samples. CHIKV = Chikungunya. 0–3 months. $p < 0.05$ was considered significant. *$p < 0.05$.
